# Clinical characteristics and prognostic factors of atrial fibrillation at a tertiary center of Pakistan – From a South-Asian perspective – A cross-sectional study

**DOI:** 10.1016/j.amsu.2021.103128

**Published:** 2021-12-09

**Authors:** Intisar Ahmed, Aiysha Nasir, Pirbhat Shams, Hunaina Shahab, Muhammad Hassan, Faryal Subhani, Ghufran Adnan, Awais Farhad, Aamir Hameed Khan, Yawer Saeed

**Affiliations:** a Section of Cardiology, Department of Medicine, The Aga Khan University Hospital, Karachi, Pakistan; b The Aga Khan University, Karachi, Pakistan

**Keywords:** Atrial fibrillation, Arrhythmia, Pakistan, South Asia

## Abstract

**Background:**

There is lack of large data from South-Asian region on atrial fibrillation and it is imperative that clinical presentation, prognostic factors, management pursued, and outcomes are known for this part of the world. Once collective evidence for the region is known, region-specific guidelines can be laid forward.

**Objectives:**

To evaluate clinical characteristics and prognostic factors of atrial fibrillation at a tertiary care center of Pakistan.

**Methods:**

This was a retrospective study conducted at a tertiary care center of Pakistan. Period of study ranged from July–December 2018. All hospitalized patients who were admitted with atrial fibrillation as a primary or associated diagnosis were enrolled.

**Results:**

A total of 636 patients were enrolled. The mean age was 68.5 ± 12 years and 49.5% (315) were male. 90.6% of the patients were admitted via emergency room. Majority (59.9%) had previously known AF and 40% developed new-onset AF during the hospital stay. Hypertension was the most common co-morbid condition (85.4%) followed by Diabetes Mellitus (40.1%). At least 9% had rheumatic heart disease. The median CHA_2_DS_2_VASc and HASBLED scores were 4 and 2 respectively. More than one-third of patients had sepsis as a primary diagnosis (36.8%). The in-hospital mortality of patients with atrial fibrillation was 6.7%. Patients with new-onset AF had higher mortality. Sepsis and stroke were independently associated with a higher mortality. There was no significant difference in median CHA_2_DS_2_VASc and HASBLED scores for patients with new-onset and previously known AF. On discharge, 83% of the eligible patients received oral anticoagulation.

**Conclusion:**

There was higher prevalence of chronic co-morbid conditions in the studied population leading to a higher CHA_2_DS_2_VASC Score. Sepsis and stroke were independently associated with higher in-hospital mortality.

## Highlights

• Sepsis and stroke are independently associated with higher in-hospital mortality in patients with atrial fibrillation.
• There is higher prevalence of chronic co-morbid conditions in the studied population.
• Hypertension is the most common co-morbid condition associated with atrial fibrillation.

## 1 Introduction

Atrial fibrillation (AF) is a supraventricular tachyarrhythmia due to an uncoordinated atrial electrical activation resulting in ineffective atrial contraction [1]. It has a complex pathophysiology. Various mechanisms have been postulated and the most widely accepted one suggests an inter-play of initiating drivers or triggers and an abnormal structural substrate (atria and pulmonary veins). Presence of structural loop (for example a diseased left atrium tissue) allows the arrhythmia to sustain by allowing multiple re-entry circuits to perpetuate [2]. AF is characterized on a surface electrocardiogram (ECG) by absence of identifiable P wave and irregular ventricular rate due to variable atrioventricular (AV) conduction [3].

AF is the most frequent sustained arrhythmia identified in clinical practice [4]. Patients with atrial fibrillation usually present with palpitations, dyspnea, and fatigue and/or chest pain. The presentation can be subclinical or asymptomatic. It is associated with a higher risk of cerebrovascular accidents (most commonly), heart failure and recurrent hospitalization. [5,6]; Atrial fibrillation can present as a primary disease or more often co-exist in patients with multiple comorbidities, such as hypertension, diabetes mellitus, coronary artery disease, cardiomyopathies, valvular heart diseases, obesity, alcohol, thyroid diseases, and chronic lung diseases [7]. Age is one of the major risk factors for developing atrial fibrillation, as much as, according to an estimate the lifetime risk of developing atrial fibrillation is 1/3rd [1]. The lifetime chance of developing atrial fibrillation increases after fifth decade [8].

With increase in life expectancy, more people now live with chronic co-morbid conditions. Hence, the prevalence of AF is estimated to rise by at least 2.5-fold by 2050 [4]. Although globally present, variation in disease burden and clinical presentation with respect to race, ethnicity and geography would not be surprising [9,10] given the difference in prevalence of co-morbid conditions across different regions of the world. In an observational study, more than 6% of the acute hospital admissions in Pakistan were due to atrial fibrillation [11]. Another study identified an incidence of 26% in patients undergoing coronary artery bypass grafting (CABG) [12].

Clinical research providing framework for guidelines over the last 2 decades are largely based on data from regions other than South-Asia [[13], [14], [15]]. There is lack of large data from South-Asian region and it is imperative that clinical presentation, prognostic factors, management pursued, and outcomes are known for this part of the world. Once collective evidence for the region is known, region-specific guidelines can be laid forward.

## 2 Methodology

**Fig. 1:**
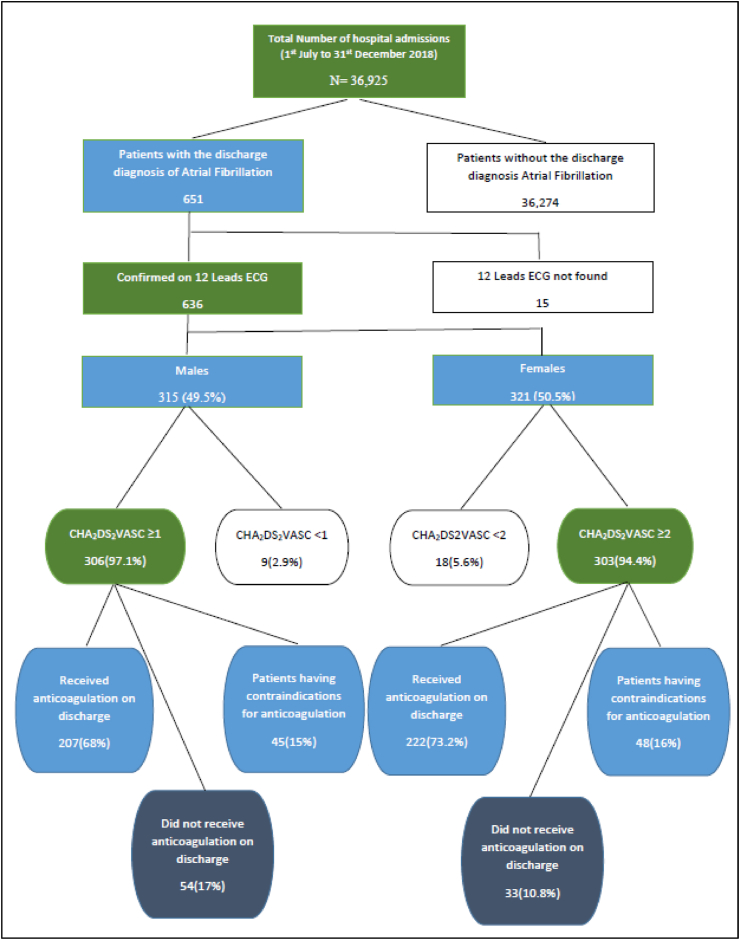
Flow Diagram representing patient recruitment.

**Study Population:** It was a retrospective observational study conducted at a tertiary care teaching hospital of the country. The study was approved by the ethical review committee of the hospital (ERC No. 019-1008-2905). Both male and females, age ≥18, who were found to have atrial fibrillation as a primary or associated diagnosis were included. Diagnosis of AF was confirmed on a 12-lead ECG. Hospital record for 651 patients meeting the inclusion criteria was retrieved from the electronic medical record system for a study period from July 1, 2018 to December 31, 2018. Fifteen patients were excluded who did not have electrocardiographic evidence of atrial fibrillation (Fig. 1).

For all the patients, basic demographic information, reason of admission, co-morbid conditions, and duration of arrhythmia were noted. Recorded comorbidities included DM, hypertension, coronary artery disease, heart failure, cardiomyopathy, valvular heart disease, chronic obstructive pulmonary disease, and hyperthyroidism. Echocardiographic data was reviewed for left atrial (LA) dimension, LA volume index and left ventricular (LV) systolic function.

Patients were labeled as diabetic if they had HbA1c > 6.5 gm/dl or were already on treatment for DM. Hypertension was defined as an average of two readings of systolic blood pressure ≥140 mmHg, a diastolic blood pressure ≥90 mmHg or if patient was already taking antihypertensive medications. Coronary artery disease was defined as ≥50% stenosis in at least one of the epicardial coronary arteries. Cardiomyopathy was defined as LV ejection fraction of ≤50%. Chronic obstructive pulmonary disease was defined as a forced expiratory volume in 1st second to forced vital capacity ratio of <70% of the predicted value. Patients were labeled hyperthyroid if they had low serum TSH and high serum concentration of free T4 or T3 as per the laboratory reference range.

**In hospital Monitoring:** All patients were followed from the time of inclusion to the time of discharge or till inpatient death. Hemodynamic parameters including heart rate and blood pressure were recorded at the time of inclusion for patients with known atrial fibrillation and at the time of onset of arrhythmia for those with new onset atrial fibrillation. Patients’ treatment records were retrieved. Complications related to atrial fibrillation, or its treatment were also recorded.

**Statistical Analysis:** After creating a database, the data was encoded. All statistical analyses were performed using the Statistical Package for Social Sciences (SPSS) version 23. Continuous variables were expressed as mean value ± standard deviation and categorical variables were expressed as frequencies and percentage. Multivariate logistic regression was done to analyze the risk factors and predictors of in-hospital mortality and p value of <0.05 was considered significant.

Ethical approval was obtained from the ethical review committee of the hospital. The work has been reported in line with the STROCSS criteria [16]. This study has been registered with clinicaltrial.gov (UIN number: NCT05082558).

## 3 Results

**Baseline characteristics:** From July–December 2018, a total of 36,925 patients were admitted to the hospital. Atrial fibrillation was present in 1.72% (636) of the patients (Fig. 1). Out of 636 patients who met the inclusion criteria, 315 (49.5%) were male with a mean age of 68.5 ± 12 years. Majority (69%) were of age ≥65 years.

Diabetes mellitus was found in 372 (58.5%) patients, hypertension in 543 (85.4%) patients, coronary artery disease in 201 (31.6%) patients and chronic kidney disease was found in 66 (10.4%) patients. A total of 117 (18.4%) had prior CVA, 39 (6.1%) patients had thyroid disorder and 57 (9%) had prior rheumatic heart disease.

Of 636 patients, 381 (59.9%) patients had previously diagnosed persistent or paroxysmal atrial fibrillation and 255 (40.1%) developed new onset atrial fibrillation during the hospital stay. Baseline characteristics of both the groups (new-onset and previously diagnosed atrial fibrillation) were as shown in Table 1. Majority of the patients (90.6%) got admitted through emergency department and sepsis/infection was the reason for hospitalization in 234 (36.8%) patients followed by heart failure in 75 (11.8%), acute coronary syndrome in 54 (8.5%) and chronic lung disease in 51 (8.0%). Only 60 (9.4%) patients were admitted electively via outpatient clinics.

**Table 1:** Baseline Characteristics of patients hospitalized with atrial fibrillation.

	Total Cohort N = 636	Paroxysmal/Persistent AF N = 381	New –onset AF N = 255
Mean Age	68.5 ± 12 Years	67.6 ± 12 Years	69.9 ± 10 Years
Patients with age <65 years	195 (30.7%)	135 (35.4%)	60 (23.5%)
Patients with age 65–74 years	237 (37.3%)	132 (34.6%)	105 (41.2%)
Patients with age ≥75 years	204 (32.1%)	114 (30.0%)	90 (35.3%)
Gender			
Male	315 (49.5%)	168 (44.1%)	147 (57.6%)
Female	321 (50.5%)	213 (55.9%)	108 (42.4%)
**Co-morbid Conditions**			
HTN	543 (85.4%)	327 (85.8%)	216 (84.7%)
DM	372 (58.5%)	223 (58.5%)	149 (58.4%)
CKD	66 (10.4%)	33 (8.7%)	33 (12.9%)
Chronic Lung Disease	51 (8.0%)		
CAD	201 (31.6%)	117 (30.7%)	84 (32.9%)
CVA	117 (18.4%)	99 (26%)	18 (7.1%)
Rheumatic Heart Disease	57 (9.0%)	57 (15%)	0 (0%)
Thyroid Disorders	39 (6.1%)	30 (7.8%)	9 (3.6%)
**Stroke and Bleeding Risk**			
CHA2DS2VASc (Median)	4 (IQR 7)	4 (IQR 7)	4 (IQR 6)
HASBLED (Median)	2 (IQR 4)	2 (IQR 4)	2 (IQR 4)
**Echocardiographic Parameters**			
Mean Ejection Fraction	48.6 ± 11%	48.1 ± 11%	49.4 ± 10%
Patients with severe LV dysfunction	72 (11.3%)	57 (15%)	15 (6%)
Mean Left Atrial Volume Index	38.8 ± 16 ml/m^2^	45 ± 17 ml/m^2^	29.5±8 ml/m^2^
Patients with Severe LA enlargement	111 (17.4%)	108 (28.3%)	03 (1.2%)
Moderate or more than moderate MS	33 (5.2%)	33 (8.6%)	0 (0%)
Moderate or more than moderate MR	159 (25%)	120 (31.5%)	39 (15.3%)
Moderate or more than moderate AS	18 (2.8%)	15 (3.9%)	03 (1.2%)
Moderate or more than moderate TR	210 (33%)	150 (39.4%)	60 (23.5%)

**Treatment:** Out of 636 patients, 489 (76.9%) patients had well controlled ventricular rate (<110beats/min) and rest of them (147) had uncontrolled ventricular rate. During the hospital stay, beta blockers were given in 465 (73.1%) patients, (relative contraindication to beta blockers was present in 162 (25.5%) patients), non-dihydropyridine calcium channel blockers in 63 (9.9%) patients. None of the patients received digoxin and none required emergency electrical cardioversion.

Median CHA_2_DS_2_VASc score in our study population was 4 with an interquartile range (IQR) of 7and median HASBLED score was 2 with an IQR of 4. Out of 321 female patients, 303 (94.4%) had a CHA_2_DS_2_VASc score of ≥2 and oral anticoagulation was prescribed to 222 (73.2%) on discharge. Among male participants, 306 (97.1%) had a CHA_2_DS_2_VASc score of ≥1 and 207 (68%) of them received anticoagulation.

Intravenous amiodarone was given in 255 (40.1%) patients, 246 of them were those with new onset AF. Mean ventricular rate of these patients was 152 ± 17 bpm and mean systolic blood pressure was 103 ± 6.4 mmHg at the time when amiodarone was started. Around 82% of the patients received intravenous amiodarone bolus (150 mg) before starting on infusion at 1 mg/min for 6 h followed by 0.5 mg/min for next 18 h. Out of 246 patients with new onset AF who received intravenous amiodarone, 60 (24.3%) reverted to sinus rhythm within 6 h of infusion and 182 (74%) were found in sinus rhythm at the end of 24 h of infusion. After intravenous infusion, oral amiodarone was continued in 142 (58.9%) of the patients during the hospital stay.

Of all the study population, 429 (67.5%) patients got oral anticoagulation on discharge and 93 (14.6%) patients did not get anticoagulation due to contraindications. Contraindications included hematuria, gastrointestinal bleed, coagulopathy, and previous intracranial hemorrhage. Novel oral anticoagulants were prescribed to 336 (52.8%) patients and 93 (14.6%) patients received vitamin K antagonist on discharge. More women received anticoagulation on discharge as compared to men (Table .2).

**Table 2:** Comparison of Males and Females with atrial fibrillation.

Variables	Males	Females	P value
Mean age (years)	69.01	68.05	0.315
Eligible for anticoagulation based on CHA2DS2VASc (≥1 in male, ≥2 for females)	306	303	0.430
Median HASBLED	2	2	0.448
Patients having contraindications to anticoagulation	45	48	0.781
**Patients received anticoagulation on discharge**	**207**	**222**	**0.033**
Mean LA volume index (ml/m2)	37.99	39.6	0.201

**Complications:** Hematuria was the most common complication observed in a total of 32 (6.6%) patients during the hospital stay, requiring transient discontinuation of anticoagulation. 5 (1.04%) of the patients developed gastrointestinal bleed requiring blood transfusion. None of the patients developed intracranial or retroperitoneal bleeding. No significant drug related side effects were found in our study population and none of the participants developed CVA during the hospital stay (Table .3).

**Table 3:** Management and complications of patients with atrial fibrillation. (n = 636).

	Total Cohort N = 636	Patients with Paroxysmal/Persistent AF N = 381	Population with New –onset AF N = 255
Anti-arrhythmic therapy			
Beta blockers	465 (73.1%)	315 (82.7%)	150 (58.8%)
Non-dihydropyridine calcium channel blockers	63 (9.9%)	27 (7%)	36 (14%)
Intravenous amiodarone	255 (40.1%)	9 (2.4%)	246 (96.4%)
**In-hospital anticoagulation**			
No. of patients received anticoagulation	480 (75.5%)	285 (74.8%)	195 (76.5%)
No. of patients having contraindication to anticoagulation	135 (21.2%)	84 (22%)	51 (20%)
**Oral anticoagulants on discharge**			
Patients received novel oral anticoagulants	336 (52.8%)	219 (57.5%)	117 (46%)
Patients received Vitamin K antagonist	93 (14.6%)	84 (22%)	9 (3.5%)
Patients having contraindication to anticoagulants	93 (14.6%)	51 (13.4%)	42 (16.5%)
**Complications**			
Significant In-hospital GI bleed	05 (1.04%)	3 (0.79%)	2 (0.78%)
In-Hospital hematuria	32 (6.6%)	25 (6.5%)	11 (4.3%)
In-Hospital death	43 (6.7%)	15 (3.9%)	28 (11%)
**Major cause of In-hospital death** (Total deaths 51)			
Sepsis/Septic shock	38 (74.5%)	13 (3.4)	25 (9.8)

During a mean hospital stay of 6.27 ± 5.22, 43 (6.7%) patients had in-hospital death and sepsis/septic shock was the major cause of in-hospital mortality. Sepsis was more common in patients with new-onset AF, resulting in higher in-hospital mortality in the group as compared to those with previously known AF.

**Predictors of In-hospital Mortality:** On multivariate logistic regression analysis, sepsis and previous cerebrovascular accident were found to be independently associated with in-hospital mortality, with odds ratio of 3.953 (95% CI 1.993–7.880) and 2.225 (95% CI 1.022–4.841) respectively. There was no association between in-hospital mortality and other co-morbid conditions such as hypertension, coronary artery disease, chronic kidney disease, gender, and thyroid disorders (Table 4).

**Table 4:** Predictors of In-hospital mortality of patients with atrial fibrillation.

Variable	Odds Ratio	95% Confidence Interval	p Value
Diabetes Mellitus	0.457	0.227–0.921	0.098
Hypertension	0.606	0.266–1.379	0.232
Coronary Artery Disease	1.638	0.778–3.450	0.194
Chronic Kidney Disease	1.526	0.580–4.016	0.392
Gender	1.541	0.761–3.117	0.229
Sepsis	3.963	1.993–7.880	0.000
Previous cerebrovascular accidents	2.225	1.022–4.841	0.044
Left Ventricular Ejection fraction	0.427	0.213–0.854	0.036
LA size (volume index)	1.432	0.716–2.864	0.394

## 4 Discussion

Major trials on drug-therapy for AF have been conducted in countries or regions other than South-Asia. Additionally, the complication spectrum of AF and AF-related drug therapy remain largely unknown for this part of the world. This brought us to the need of studying AF at our center. Our study highlights the clinical characteristics, drug prescription practice for AF patients and reveals AF-related complications.

The in-hospital prevalence of AF in our study population was 1.72% which is relatively lower than the one reported in a study from Denmark that looked at hospital admissions data from 1997 to 2009 and reported a prevalence of 6.8% in hospitalized patients [17]. Similarly, a study from Kuwait over a 5-month period showed 4.24% of acute hospital admissions were associated with AF [18]. Of note, the overall AF prevalence is lower despite a high prevalence of risk factors for AF such as hypertension and diabetes mellitus. On the other hand, prevalence of new-onset AF was higher (40%) than what had been previously reported in the literature. Allen J Walkey et al. reported that 6.64% of patients developed new onset atrial fibrillation during hospitalization [19]. Haq et al. reported AF in at least 27% of patients [11]. Possible explanation includes a sizeable number of our patients with infection and sepsis (30.6%), which is one of the major predisposing factors for new onset atrial fibrillation [20]. In a prospective study conducted in Pakistan over span of two months, 6.5% of hospitalized patients had atrial fibrillation in background of higher incidence of rheumatic heart disease [11]. On the other hand, we had a relatively lower prevalence of rheumatic heart disease. This could be due to the differences in urbanicity of the two cities.

Our study showed that patients with new onset AF did not differ from patients with previously known AF in terms of baseline characteristics and co-morbid conditions such as DM and hypertension. It is quite possible that patients with new onset AF previously had asymptomatic or paroxysmal AF which went undocumented due to the lack of hospital encounter.

In our study population, 83% of the eligible patients (CHA_2_DS_2_VASc ≥1 in males and ≥2 in females) received anticoagulation on discharge. Haq et al. reported that only 48% of the study population was discharged on oral anticoagulation [11]. Moreover, in GRASP-AF registry, more than one third of the patients in United Kingdom, who were eligible for anticoagulation and had risk of stroke did not receive anticoagulation [20]. This higher rate of anticoagulation prescription could be because all the patients with AF were reviewed by cardiologist- or an internist-on call and CHA_2_DS_2_VASc risk score was calculated for each patient. NOACs (novel oral anticoagulants) were the most used anticoagulant in 336 (78%) of patients.

Our study population had a higher CHA_2_DS_2_VASc score with a median score of 4 (No difference was observed between new onset and previously known AF patients). Tischer et al. from Germany reported a mean CHA_2_DS_2_VASc of 3.04 ± 1.42 in 150,408 AF patients admitted to a tertiary care hospital [21]. With the mean age being comparable between the two studies (68 ± 12 year and 67.6 ± 13.6 year), the higher CHA_2_DS_2_VASc score can be explained by the higher prevalence of co-morbid conditions such as hypertension (85%), DM (58.5%) and prior CVA (18.4%). The relatively higher prevalence of prior stroke in the study population points towards late diagnosis once complication of AF had already happened. Considering higher prevalence of risk factors associated with AF in the region, it is imperative that an effort for early recognition of AF is made at a community level.

Our study highlighted a higher conversion-to-sinus rate (74%) with use of amiodarone along with a relatively lower rate of recurrence during the same hospital stay (7.4%). In a study by Mitric et al., 95% of the patients with new onset AF cardioverted to sinus rhythm with amiodarone, however AF recurred in 51.4% patients [22]. In a study by Dursun et. Al., 81 out of 106 patients converted to normal sinus rhythm within 24 h [23]. In another study by Kanji S. et al., 73% of the patients with new onset atrial fibrillation, cardioverted to sinus rhythm within 24 h and around 18% developed recurrence [24]. We can conclude from our study that use of amiodarone had better efficacy in the studied population.

In our study population, moderate to severe mitral stenosis was seen only in 5.2% of patients and only 9% has a diagnosis of rheumatic heart disease (RHD). These findings are quite different from the Haq et al. study which showed 27% of patient with rheumatic heart disease [11]. This may well represent the changing paradigm in the incidence of RHD in urban Pakistani population in the last 10 years. We have also found that 57% (263) patients had left atrial enlargement and 111 (17.5%) had severe left atrial enlargement. Mean LA volume index was >38ml/m2 in our study population, which is relatively high. This is in correlation with age of our study population and presence of multiple comorbidities.

In our study population, majority of patients with new onset atrial fibrillation had underlying sepsis. The in-hospital mortality was more than 6% in our population which is higher than the comparative studies. In a population-based study from Korea, patients with AF had four times higher mortality as compared to general population and cerebrovascular accident (CVA) was the most common cause of death in patients with AF [25]. In a systemic review by Sumeet Gandhi, sepsis with new onset atrial fibrillation was associated with increased hospital stay and increased in-hospital mortality [26]. This is consistent with our data as we have also identified that CVA and sepsis were independently associated with in-hospital mortality.

To our knowledge, this is one of the largest hospital-based study on AF from Pakistan. This study identifies important information regarding the clinical characteristics and management of AF patients in Pakistan.

## 5 Limitations

This is a single centered retrospective observational study and may not represent the entire population of the country. There was no control group in the study, and it was not possible to establish the causation of atrial fibrillation with mortality. Long-term follow-up was not available to assess the complications of AF and drug compliance.

## 6 Conclusion

The studied population had a higher CHA_2_DS_2_VASc score and multiple comorbid conditions. Non-valvular atrial fibrillation was much more common than the valvular atrial fibrillation. Sepsis and prior cerebrovascular accidents were independently associated with in-hospital mortality in our study population. One-fifth of hospitalized patients with AF had history of CVA. However, the anticoagulation rate on discharge among eligible patient was around 83%. There is a need to recognize patients at risk of developing atrial fibrillation and its associated complications. A population-based study is required to confirm above findings. This shall lead to the foundation of local guidelines for the management of AF.

## Ethical approval

Ethical review committee of the Aga Khan University Hospital.

## Sources of funding for your research

No funding acquired for this study.

ERC Number: 019-1008-2905.

## Author contribution

IA: Study conceptualization, data management and analysis and manuscript writing.

AN, PS, MH, FS collected the data and contributed to the data analysis and literature search.

HS, GA, AF contributed to the manuscript review and literature search.

AH: critical appraisal and manuscript review.

YS: Supervised from conceptualization to final execution and manuscript writing.

## Consent

Consent not applicable as no direct intervention or interaction with human subjects.

## Registration of research studies

NOT APPLICABLE.

1. Name of the registry: clinicaltrial.gov.

2. Unique Identifying number or registration ID: NCT05082558.

3. Hyperlink to your specific registration (must be publicly accessible and will be checked): https://register.clinicaltrials.gov/prs/app/action/SelectProtocol?sid=S000BBXV&selectaction=Edit&uid=U0005QOF&ts=2&cx=-3zumv5.

## Guarantor

Dr. Yawer Saeed.

## Declaration of competing interest

None of the authors has any conflict of interest to reveal.
